# Calicivirus Non-structural Proteins: Potential Functions in Replication and Host Cell Manipulation

**DOI:** 10.3389/fmicb.2021.712710

**Published:** 2021-07-14

**Authors:** Elena Smertina, Robyn N. Hall, Nadya Urakova, Tanja Strive, Michael Frese

**Affiliations:** ^1^Commonwealth Scientific and Industrial Research Organization, Health and Biosecurity, Canberra, ACT, Australia; ^2^Faculty of Science and Technology, University of Canberra, Canberra, ACT, Australia; ^3^Centre for Invasive Species Solutions, Canberra, ACT, Australia; ^4^Department of Medical Microbiology, Leiden University Medical Center, Leiden, Netherlands

**Keywords:** RNA virus, *Caliciviridae*, non-structural proteins, viroporin, replication

## Abstract

The *Caliciviridae* are a family of viruses with a single-stranded, non-segmented RNA genome of positive polarity. The ongoing discovery of caliciviruses has increased the number of genera in this family to 11 (*Norovirus*, *Nebovirus*, *Sapovirus*, *Lagovirus*, *Vesivirus*, *Nacovirus*, *Bavovirus*, *Recovirus*, *Salovirus*, *Minovirus*, and *Valovirus*). Caliciviruses infect a wide range of hosts that include fishes, amphibians, reptiles, birds, and marine and land mammals. All caliciviruses have a genome that encodes a major and a minor capsid protein, a genome-linked viral protein, and several non-structural proteins. Of these non-structural proteins, only the helicase, protease, and RNA-dependent RNA polymerase share clear sequence and structural similarities with proteins from other virus families. In addition, all caliciviruses express two or three non-structural proteins for which functions have not been clearly defined. The sequence diversity of these non-structural proteins and a multitude of processing strategies suggest that at least some have evolved independently, possibly to counteract innate and adaptive immune responses in a host-specific manner. Studying these proteins is often difficult as many caliciviruses cannot be grown in cell culture. Nevertheless, the study of recombinant proteins has revealed many of their properties, such as intracellular localization, capacity to oligomerize, and ability to interact with viral and/or cellular proteins; the release of non-structural proteins from transfected cells has also been investigated. Here, we will summarize these findings and discuss recent *in silico* studies that identified previously overlooked putative functional domains and structural features, including transmembrane domains that suggest the presence of viroporins.

## Introduction

The *Caliciviridae* family of RNA viruses currently includes 11 genera, i.e., *Norovirus*, *Nebovirus*, *Sapovirus*, *Lagovirus*, *Vesivirus*, *Nacovirus*, *Bavovirus*, *Recovirus*, *Salovirus*, *Minovirus*, and *Valovirus* (Desselberger, 2019; Vinjé et al., 2019). Viruses of the genera *Norovirus*, *Nebovirus*, *Sapovirus*, *Lagovirus*, *Recovirus*, and *Valovirus* are enteric viruses of mammals. Some of these viruses are associated with severe gastroenteritis or systemic disease, while others cause only mild or asymptomatic infections. Noroviruses cause an estimated 684 million gastroenteritis episodes and 200,000 deaths annually imposing a significant economic burden (Kirk et al., 2015; Pires et al., 2015; Bartsch et al., 2016). Human norovirus and sapovirus infections can also lead to chronic disease and are often associated with severe complications, especially in the elderly, very young, and immunocompromised patients (Petrignani et al., 2018; Wright et al., 2020). Neboviruses are enteric pathogens of cattle in which mortality rates reach up to 30% (Alkan et al., 2015). *Tulane virus* (genus *Recovirus*; Farkas et al., 2008) was isolated from stool samples of rhesus macaques; another recovirus (Bangladesh/289/2007) was later discovered from human patients with diarrhea in Bangladesh (Smits et al., 2012). Remarkably, and in contrast to other human caliciviruses, *Tulane virus* easily propagates in cell culture (Farkas, 2015), which promises to turn this newly discovered virus into an important model for enteric caliciviruses. Valoviruses were first isolated from the feces of asymptomatic farmed pigs; these viruses were named St-Valérien-like viruses and found to be closely related to noroviruses and recoviruses (L’Homme et al., 2009). Bavoviruses and nacoviruses were recovered from feces and intestinal contents of poultry – chickens, turkeys, and geese (Wolf et al., 2012; Liao et al., 2014). Rabbit caliciviruses (RCVs) and hare caliciviruses are enteric lagoviruses. The name “lagovirus” refers to the narrow host range of these viruses; they infect only members of the order Lagomorpha, e.g., *Oryctolagus* (European rabbit), *Lepus* (hares and jackrabbits), and *Sylvilagus* (cottontail rabbits). RCVs such as RCV-A1 usually cause asymptomatic infections in rabbits in contrast to many other lagoviruses that have been discovered to date (Strive et al., 2010). Pathogenic lagoviruses, including *Rabbit hemorrhagic disease virus* (RHDV) and *European brown hare syndrome virus* (EBHSV), are hepatotropic and cause a peracute hepatitis with mortality rates approaching 100% (Abrantes et al., 2012; Hall et al., 2017). Vesiviruses are the caliciviruses with the widest host range; so far, viruses have been isolated from cats (*Feline calicivirus*, FCV), pigs (*Vesicular exanthema of swine virus*, VESV), and seals (*San Miguel sea lion virus*-8, SMSV-8; Oglesby et al., 1971; Radford et al., 2007; Neill, 2014). Infection with vesiviruses can cause multiple organ failure, vesicular lesions, and respiratory and reproductive system diseases, depending on virus and host species (Radford et al., 2007). Saloviruses and minoviruses are viruses that infect fishes. *Atlantic salmon calicivirus* (ASCV, genus *Salovirus*) was isolated from heart tissue of farmed Atlantic salmons with symptoms of heart and skeletal muscle inflammation (Mikalsen et al., 2014). *Fathead minnow calicivirus* (FHMCV, genus *Minovirus*) was first identified in diseased fathead minnows with widespread hemorrhaging; however, all analyzed fish samples showed a co-infection with fathead minnow picornavirus. Thus, further studies are needed to elucidate whether FHMCV is associated with hemorrhagic disease or requires a co-infection to cause the disease (Mor et al., 2017).

**Figure 1:**
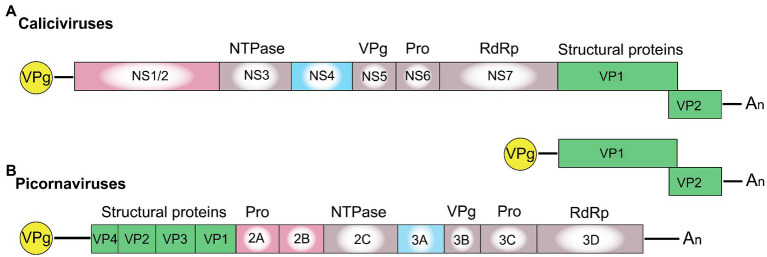
Schematic representation of the genome organization for a typical **(A)** calicivirus and **(B)** picornavirus. Untranslated and translated sequences are depicted as lines and boxes, respectively. Structural (capsid) proteins are shown in green (note that VP1 and VP2 in lagoviruses are usually referred to as VP60 and VP10, respectively); the signature proteins of the picornavirus-like superfamily are shown in gray, namely the helicase (NTPase), VPg, protease (Pro), and the RNA-dependent RNA polymerase (RdRp); the non-structural calicivirus proteins NS1/2 and their positional homologs in picornaviruses (2A, 2B) are shown in pink; and the non-structural calicivirus protein NS4 and its homolog in picornaviruses (3A) are shown in blue. Covalently linked VPg proteins at the 5' end are shown in yellow, and An represents the poly(A) tail at the 3' end.

*Picornaviridae* and *Caliciviridae* are closely related families of the order *Picornavirales*, which comprises non-enveloped viruses with a positive-sense RNA genome. Both virus families direct host cells to synthesize a polyprotein that is cleaved by viral proteases, a process that, in some caliciviruses, is assisted by cellular proteases (Thumfart and Meyers, 2002; Sosnovtsev et al., 2006). In the case of caliciviruses, mature non-structural proteins include the RNA-dependent RNA polymerase (RdRp), a 3C-like protease, VPg (virion protein, genome-linked), a helicase (NTPase), and several poorly characterized non-structural proteins that can be termed NS1, NS2, and NS4. The overall gene organization of caliciviruses resembles that of picornaviruses with one major difference (Figure 1). In caliciviruses, the coding sequence for the capsid proteins is located at the 3'-end, while in picornaviruses, the capsid genes precede the polyprotein and are the first to be translated. Thus, the positional homologs of the calicivirus non-structural proteins NS1, NS2, and NS4 in picornaviruses are 2A, 2B, and 3A, respectively.

The genetic material of all caliciviruses consists of two types of positive-sense, single-stranded RNA molecules: full-length genomic and subgenomic RNAs (by contrast, picornaviruses produce only full-length genomic RNA). Calicivirus particles contain a single copy of genomic RNA (ca 7.5 kb) or one or more copies of a subgenomic RNA (ca 2 kb; Ehresmann and Schaffer, 1977; Meyers et al., 1991a,b). Both RNAs have a 5' end that is covalently linked to VPg, a polyadenylated 3' end, and coding sequences in two or more partially overlapping open reading frames (ORFs) that are flanked by untranslated regions (UTRs). While the genomic full-length RNA encodes all structural and non-structural proteins, the subgenomic RNA encodes only the structural proteins VP1 and VP2. In caliciviruses, the subgenomic RNA ensures efficient particle formation through the synthesis of additional capsid proteins (Miller and Koev, 2000). It is tempting to speculate that picornaviruses do not require subgenomic RNAs as the positioning of the coding sequences for the structural proteins at the 5' end increases protein output. Why caliciviruses have evolved a different coding strategy is not clear; however, having an additional RNA molecule for the enhanced expression of structural proteins may allow for a more sophisticated control of protein production (Meyers, 2003).

**Figure 2:**
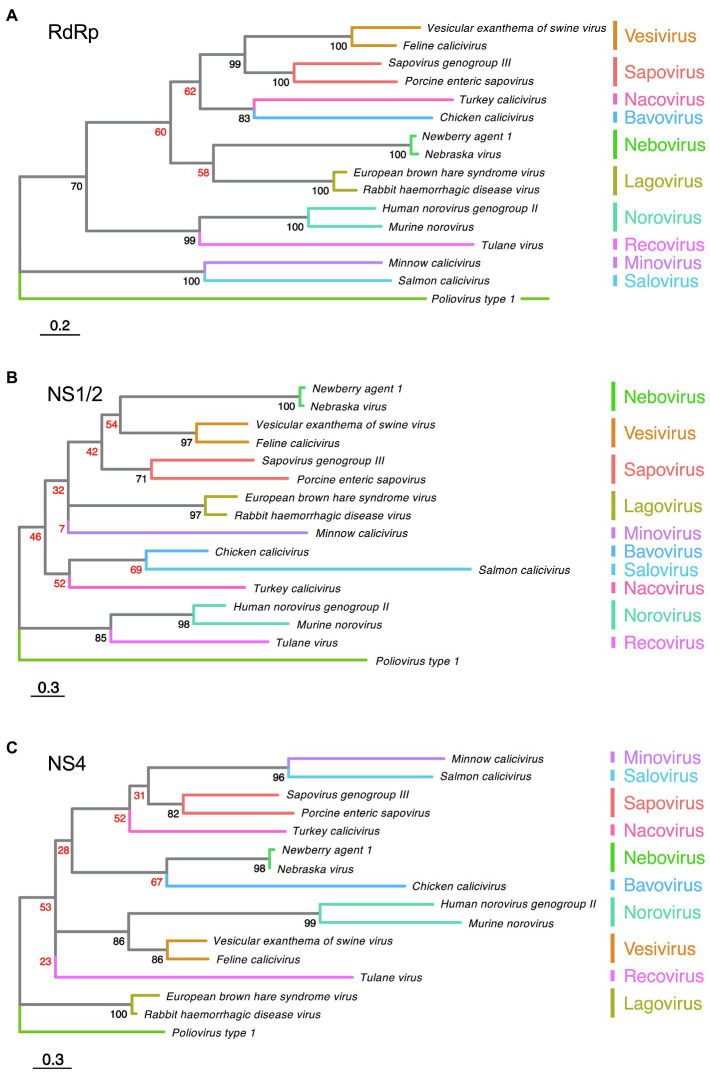
Phylogenetic analysis of calicivirus protein sequences. Maximum likelihood phylogenies were inferred for amino acid sequences of **(A)** RdRp, **(B)** NS1/2, and **(C)** NS4. First, the amino acid sequences of the complete ORF1 coding region of representative published calicivirus sequences were aligned using MAFFT (Katoh et al., 2002). The alignment was then curated with trimAl (Capella-Gutiérrez et al., 2009). The RdRp, NS1/2, and NS4 coding regions were extracted from this complete ORF1 alignment and phylogenies were inferred individually for each gene using IQ-TREE (Nguyen et al., 2015). Phylogenies were rooted using *Poliovirus type 1* (GenBank accession NC_002058). The following sequences were chosen for calicivirus genera: *Vesicular exanthema of swine virus* (VESV; NC_002551), *Feline calicivirus* (FCV; NC_001481), *Sapovirus genogroup III* (MG012434), *Porcine enteric sapovirus* (NC_000940), *Turkey calicivirus* (NC_043516), *Chicken calicivirus* (NC_033081), *Newbury agent 1* (NC_007916), *Nebraska virus* (NC_004064), *Rabbit haemorrhagic disease virus* (NC_001543), *European brown hare syndrome virus* (EBHASV; NC_002615), *Tulane virus* (NC_043512), *Human norovirus genogroup II* (NC_039477), *Murine norovirus* (MNV; NC_008311), *Minnow calicivirus* (NC_035675), and *Salmon calicivirus* (NC_024031). The tree is drawn to scale, with branch lengths measured in the number of substitutions per site. Ultrafast bootstrap values are shown for each node. Low confidence bootstrap values (<70) are highlighted in red.

The functions of the calicivirus RdRp, protease, helicase, and VPg were identified based on sequence similarities to homologous proteins from picornaviruses and other positive-sense single-stranded RNA viruses (Neill, 1990; Lambden and Clarke, 1995; Clarke and Lambden, 1997). The presence of conserved motifs and domains in these proteins often indicates the function (e.g., almost all RNA polymerases have a GDD motif in their active site). The RdRp replicates the viral genome, a process that, due to the lack of proof reading, constantly generates considerable genetic diversity. Template switching can further increase genetic diversity; in calicivirus-infected cells, this occurs relatively frequently, and most commonly at the junction of RdRp and structural protein coding sequences (Mahar et al., 2013). The calicivirus protease, also referred to as the 3C-like protease after its counterpart in picornaviruses, participates in the proteolytic cleavage of the viral polyprotein (Boniotti et al., 1994). The calicivirus helicase unwinds double-stranded RNA intermediates in an ATP-dependent reaction during viral replication (similar to homologous proteins in other viruses). However, the calicivirus helicase has additional functions. It acts as an RNA chaperone that remodels structured RNA in an ATP-independent manner (Li et al., 2017), and it facilitates the formation of vesicular structures that house the replication complexes (Cotton et al., 2016). The VPg protein is usually listed among the non-structural proteins, but as it is covalently bound to the 5' end of both genomic and subgenomic RNAs and is therefore present in mature virus particles, it could arguably be categorized as a structural protein. In infected cells, the VPg serves as a primer for the replication of the viral genome and plays a critical role in the initiation of translation (Herbert et al., 1997; Goodfellow, 2011). The functions of the remaining non-structural proteins (i.e., NS1/2, NS1, NS2, and NS4) are more challenging to determine, as they lack sequence homology to other proteins. Even within the *Caliciviridae* family, the sequence diversity is so great that non-structural protein sequences cannot be used to produce meaningful phylogenetic trees, except for sequences from closely related viruses (Figures 2B,C). Highly conserved RdRp sequences, in contrast, are much more suitable for phylogenetic analyses (Koonin, 1991; Wolf et al., 2018; Figure 2A). The location of the non-structural protein genes in the viral genome, however, is rather conserved. In all caliciviruses, the coding sequence of NS1/2 is located at the 5' end, while the NS4 sequence follows the helicase sequence. Some, but not all, NS1/2 proteins undergo proteolytic cleavage. In vesiviruses, lagoviruses, neboviruses, and sapoviruses, the NS1/2 precursor protein is efficiently cleaved by viral and/or host cell proteases, generating the proteins NS1 and NS2. In other viruses, the cleavage efficiency is less clear, and more stable precursor proteins may exist.

Although function(s) of the non-structural proteins NS1/2 and NS4 remain elusive, recent studies suggest that mutations in their coding sequences may influence tissue tropism, virulence, and epidemiological fitness. For example, Mahar et al. (2021) provide evidence for a role of NS proteins in epidemiological fitness, while investigating the evolution of lagoviruses. The recent introduction of the highly pathogenic RHDV2 to Australia quickly led to the emergence of recombinant lagoviruses that contain the capsid genes of RHDV2 and the non-structural protein coding sequences of non-pathogenic RCV strains that had been circulating in Australian rabbits for decades. These recombinants are hepatotropic and highly pathogenic (as is the parental RHDV2), suggesting that virulence and tropism is conferred by the structural genes. Furthermore, the recombinant strains quickly replaced RHDV2 despite having an identical or near-identical capsid protein, which suggests that non-structural proteins are important drivers of epidemiological fitness (Mahar et al., 2021). It would be interesting to extend these studies to explore which non-structural protein or which combination of non-structural proteins is responsible for the evolutionary success of these recombinant lagovirus strains in Australia. Non-structural proteins have also been shown to influence the tissue tropism in some caliciviruses. For example, in *Murine norovirus* (MNV), a single amino acid substitution in NS1 is associated with better virus growth and persistent infection of the proximal colon. A non-persistent strain becomes persistent with a single change of aspartic acid to glutamic acid (D93E) in NS1 (Nice et al., 2013). Clearly, evidence is mounting for a role of the non-structural proteins as key determinants of pathogenicity and epidemiological fitness. In this review, we summarize the current knowledge of these calicivirus proteins.

## Non-Structural Protein Processing and Secretion

**Figure 3:**
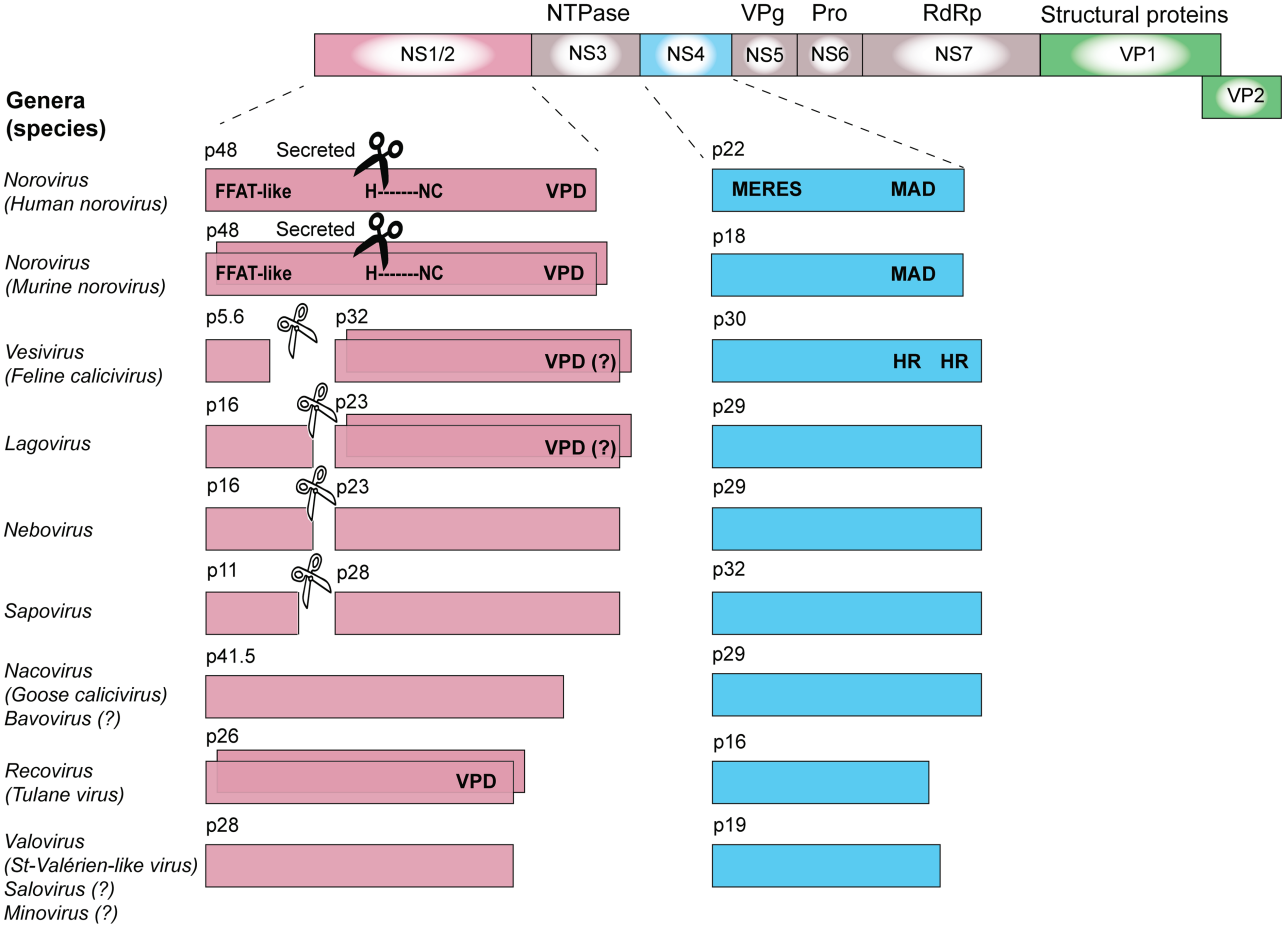
Calicivirus non-structural protein processing, oligomerization, and domains. The coding sequences for non-structural proteins are shown in pink (NS1/2), blue (NS4), or gray (all other non-structural proteins), while coding sequences for structural proteins are shown in green (VP1 and VP2). The ability of some proteins to oligomerize is illustrated by staggered boxes. Black scissors indicate the presence of a caspase-3 cleavage site; white scissors indicate a viral 3C-like protease cleavage site. VPD, viroporin domain; HR, hydrophobic region; MAD, membrane-associated domain; H-NC, H-box and NC motif (asparagine-cysteine); MERES, mimic of an endoplasmic reticulum (ER) export signal; and FFAT, phenylalanine-phenylalanine acidic tract. Genes and gene products are drawn to scale.

The polyprotein of all caliciviruses is cleaved by the 3C-like virus protease into the non-structural proteins NS1/2, helicase, NS4, VPg, 3C-like protease, and the RdRp (Figure 3). In RHDV, FCV, and human sapovirus infected cells, the NS1/2 precursor is also cleaved by the 3C-like protease (Wirblich et al., 1996; Sosnovtsev et al., 2002; Oka et al., 2005). In contrast, the norovirus NS1/2 precursor is not processed further by the 3C-like protease (Liu et al., 1996). However, when the processing of a recombinant MNV NS1/2 precursor was analyzed *in vitro*, it was discovered that a cellular protease, caspase-3, cleaves the protein (Sosnovtsev et al., 2006). Subsequently, a cleavage site homolog was identified in human noroviruses and it was discovered that both the human norovirus and MNV secrete NS1 after the precursor has been cleaved (Lee et al., 2019). Cleavage occurs only at a late stage of the viral life cycle (18–22 h post-infection) and is concurrent with activation of apoptosis (Robinson et al., 2019). Moreover, this process is required for intestinal tropism and virus persistence. A persistent MNV strain with a deleted caspase-3 cleavage site replicated less efficiently in the ileum (10-fold decrease) and was rarely detected in feces compared with the corresponding wild-type strain (Robinson et al., 2019). Interestingly, the “secretion” of NS1 is insensitive to brefeldin A, an inhibitor of trafficking from the endoplasmic reticulum (ER) to the Golgi (Lee et al., 2019). This suggests that NS1 leaves infected cells through an unconventional pathway that still awaits characterization.

## Non-Structural Protein Oligomerization

The ability to oligomerize has been demonstrated for several non-structural proteins, including the NS1/2 of MNV and *Tulane virus*, NS2 of FCV, and NS2 of RHDV (Table 1). One of the best-studied examples is the NS2 (p23) protein of RHDV, which was shown to oligomerize using co-translocation and cross-linking assays (Urakova et al., 2015). Briefly, rabbit kidney (RK)-13 cells were transfected with expression plasmids that encoded either a NS2 protein with a nuclear localization sequence (NLS) and a FLAG-tag or a NS2 protein with a myc-tag but no NLS. When expressed separately, only the NS2 protein with an NLS was transported to the nucleus. Upon co-expression, however, both proteins were detected in the nucleus, suggesting that NS2 proteins form dimers or higher order oligomers. This protein-protein interaction was confirmed in cross-linking experiments; subsequent Western blot analysis revealed a band of dimeric NS2 (Urakova et al., 2015). More evidence for the ability of NS2 proteins to oligomerize has been observed with FCV and *Tulane virus*. In the case of FCV, NS2-NS2 dimers were detected in lysates from transfected cells after proteins were separated under non-reducing conditions and analyzed by Western blotting (Kaiser, 2006). In the case of *Tulane virus*, NS1/2 oligomers were detected by Western blotting of unboiled lysates from transfected cells that were also separated under non-reducing conditions (Strtak et al., 2019). Taken together, these findings indicate that the oligomerization domain in NS1/2 resides in the NS2 moiety of the protein. Interestingly, recent reports suggest that the NS2 part of the NS1/2 protein of noroviruses and *Tulane virus* possesses viroporin activity, for which oligomerization is essential (Strtak et al., 2019).

**Table 1:** Cellular localization and functional properties of calicivirus non-structural proteins.

Protein name	Intracellular localization	Major functions/features	Reference
*Human norovirus* (genus *Norovirus*)			
NS1	Extracellular	NS1 is secreted and counteracts innate immune responses mediated by IFN-λ	Lee et al., 2019
NS1/2	Golgi	Golgi disassembly; inhibition of cellular protein secretion	Ettayebi and Hardy, 2003 Fernandez-Vega et al., 2004
NS4	ER/Golgi	Golgi disassembly; inhibition of cellular protein secretion; formation of membranous web (replication factories)	Sharp et al., 2010 Doerflinger et al., 2017
*Murine norovirus* (genus *Norovirus*)			
NS1	Extracellular	NS1 is secreted and counteracts innate immune responses mediated by IFN-λ	Lee et al., 2019
NS1/2	ER	NS1/2 dimerization; NS1/2 cleavage is associated with persistence and apoptosis	Hyde and Mackenzie, 2010 Baker et al., 2012 Lee et al., 2019 Robinson et al., 2019
NS4	Golgi, endosomes	Golgi disassembly; moderate inhibition of cellular protein secretion	Hyde and Mackenzie, 2010 Sharp et al., 2012
*Feline calicivirus* (genus *Vesivirus*)			
NS2	ER	Dimerization; formation of membranous web (replication factories)	Kaiser, 2006 Bailey et al., 2010
NS4	ER	Formation of membranous web (replication factories); counteracts IFN response	Bailey et al., 2010 Tian et al., 2020
*Rabbit haemorrhagic disease virus* (genus *Lagovirus*)			
NS1	Nucleus and cytoplasm	Unknown	Urakova et al., 2015
NS2	Cytoplasm (ER?)	Dimerization	
NS4	Cytoplasm	Unknown	
*Tulane virus* (*genus Recovirus*)			
NS1/2	ER	Oligomerization; viroporin formation	Strtak et al., 2019

## Membrane Association of Non-Structural Proteins

All positive-sense RNA viruses, including caliciviruses and picornaviruses, are known to manipulate host cell membranes to create a “virus-friendly” environment (Romero-Brey and Bartenschlager, 2014). A re-organization of the production and trafficking of cellular membranes has many consequences, e.g., interferons (IFNs) may no longer be secreted and major histocompatibility complex (MHC) molecules may no longer reach the plasma membrane. This allows viruses to become “invisible” to the innate and adaptive immune systems. In addition, re-directing cellular membrane trafficking enables the formation of new intracellular compartments for virus replication. In picornaviruses, the ability of 2A, 2B, and 3A to manipulate cellular membranes is well characterized (Doedens and Kirkegaard, 1995; Neznanov et al., 2001). It is tempting to speculate that these proteins are functional homologs of the calicivirus proteins NS1/2 and NS4. In the following, we will discuss findings that support this hypothesis.

### Manipulation of Cellular Membrane Trafficking

Poliovirus (genus *Enterovirus*) proteins 2B and 3A co-localize with ER and/or Golgi membranes in transfected cells, manipulating cellular membrane networks. For example, 3A is known to disrupt the Golgi architecture by inhibiting the vesicle transport from ER to Golgi (Teterina et al., 2011). Another consequence of 3A expression is the formation of vesicles that facilitate viral replication, most likely through the recruitment of ER-derived membranes (Cho et al., 1994; Suhy et al., 2000). Similarly, the calicivirus non-structural proteins NS1/2 and NS4 seem to localize either to Golgi or ER membranes (Table 1). In some cases, however, the intracellular localization was not determined in great detail, i.e., it is often not known, where exactly a particular non-structural protein is localized in transfected cells (let alone during genuine virus replication).

The human norovirus protein NS4 localizes to the ER and Golgi (Table 1). This protein contains an YХФESDG motif (where X is any amino acid and Ф is a hydrophobic amino acid residue), mimicking cellular ER export signals that typically contain a short YXXФ motif (Nishimura and Balch, 1997). This norovirus motif was thus named mimic of an endoplasmic reticulum export signal (MERES; Sharp et al., 2012). It is therefore not surprising that NS4 antagonizes trafficking from the ER to Golgi (Sharp et al., 2012). By using a recombinant alkaline phosphatase as a reporter, it was shown that norovirus NS4 hijacks COPII vesicles and inhibits protein secretion (Fernandez-Vega et al., 2004; Sharp et al., 2010). Similar but less prominent effects were demonstrated for the NS4 protein of MNV, which lacks a MERES motif, but nevertheless localizes with Golgi membranes (Sharp et al., 2012). The interaction of the human norovirus NS4 with Golgi membranes occurs *via* a hydrophobic membrane association domain (MAD) that contains an amphipathic α-helix capable of membrane insertion (Sharp et al., 2010). When Doerflinger et al. (2017) used light and electron microscopy to study the impact of recombinant NS4 proteins on vesicle formation, they found that NS4 is sufficient to induce the formation of single and double membrane vesicles. Taken together, NS4 seems to play a key role in the manipulation of protein trafficking and the formation of replication factories.

Inhibiting protein secretion in norovirus-infected cells is not restricted to NS4. The human norovirus protein NS1/2 also disrupts the Golgi and shows a vesicular localization pattern in transfected cells (Ettayebi and Hardy, 2003). To study the effect of recombinant NS1/2 expression, researchers traced the fate of the vesicular stomatitis virus (VSV) glycoprotein G in transfected cells. In the absence of NS1/2, the VSV G protein was transported to the cellular surface. In cells that also expressed the human norovirus NS1/2, VSV G was no longer detectable on the cell surface, suggesting a disruption of the vesicular transport. Instead, VSV G was found to partially co-localize with NS1/2 (Ettayebi and Hardy, 2003). A yeast two-hybrid screen revealed an interaction of human NS1/2 with the vesicle-associated membrane protein VAP-A (Ettayebi and Hardy, 2003). Since VAP-A plays an important role in the ER-to-Golgi vesicle trafficking (Weir et al., 1998, 2001), Ettayebi and Hardy (2003) hypothesized that the interaction between NS1/2 and VAP-A contributes to the inhibition of secretory pathways. Subsequently, this interaction was found to be dependent on a motif in NS1/2 that mimics the cellular phenylalanine-phenylalanine-acidic-tract (FFAT) motif (Figure 3; McCune et al., 2017). FFAT motifs are present in many cellular proteins that bind VAP-A (Kaiser et al., 2005), which further supports the idea that NS1/2 proteins of human noroviruses inhibit secretory pathways through an interaction with VAP-A. A similar protein-protein interaction has been found for NS1/2 of MNV and VAP-A (McCune et al., 2017). Taken together, these findings suggest that manipulating VAP-A might be an important strategy in the calicivirus life cycle. Future studies that investigate protein-protein interactions of other NS1/2 proteins (or their cleavage products) will reveal whether all caliciviruses rely on this strategy.

### Formation of Membrane-Associated Replication Complexes

The key protein in calicivirus replication is the RdRp (also referred to as NS7, e.g., in the case of noroviruses). Calicivirus RdRps are well-studied; crystal structures have been determined for *Norwalk virus*, MNV, *Sapporo virus*, and RHDV (Ng et al., 2002, 2004; Fullerton et al., 2007; Lee et al., 2011). As with all viral RdRps, the tertiary structure of the calicivirus RdRps resembles the shape of a right hand, with distinctive domains named “fingers,” “palm,” and “thumb.” Within these domains, seven highly conserved short motifs have been identified (motifs “A” to “G”), each with a distinctive function in RNA replication (reviewed in Te Velthuis, 2014; Deval et al., 2017). Moreover, an additional 8th motif (motif “I”) has recently been identified in both calicivirus and picornavirus RdRps, although a specific function has not yet been assigned to this motif (Smertina et al., 2019). VPg is another viral protein that is directly involved in viral genome replication. The RdRp and its protease-polymerase (Pro-Pol) precursor nucleotidylate VPg (Belliot et al., 2008; Han et al., 2010); nucleotidylated VPg acts as a primer for genomic and possibly antigenomic RNA synthesis (summarized in Smertina et al., 2019). Consequently, all genomic and subgenomic RNAs are covalently linked at the 5' end to a VPg protein, which enables a “cap”-independent translation of viral RNAs (Goodfellow, 2011; Leen et al., 2016). Apart from the RdRp and VPg, other non-structural viral proteins have been found at the site of RNA replication (Green et al., 2002; Hosmillo et al., 2019), but how these proteins assist in RNA replication is less clear. Potential roles for these proteins, e.g., in the recruitment of membranes to anchor and shield the RNA replication machinery, are discussed below.

The NS2 and NS4 proteins of FCV are predicted to contain membrane-spanning hydrophobic protein domains, suggesting an association with membranes (Bailey et al., 2010). This might explain the ER localization of these proteins in immunofluorescence studies (Table 1). Immunofluorescence and electron microscopy studies further revealed that NS2 and NS4 of FCV cause a dramatic reorganization of the ER in transiently transfected Crandell Reese feline kidney (CRFK) cells (Bailey et al., 2010). Furthermore, the manipulation of the intracellular membrane traffic resulted in the formation of vesicles associated with virus replication (Bailey et al., 2010). Similar observations were made in FCV-infected 293T cells; however, an impairment of cellular secretory functions was not detected (Bailey et al., 2010). An association of FCV NS2 and NS4 with viral replication complexes has long been postulated based on protein-protein interaction. Green et al. (2002) isolated active replication complexes from FCV-infected cells and identified the presence of NS2, NS4, helicase, capsid proteins, the polymerase precursor protein Pro-Pol, and the NS4-VPg precursor. These results were later confirmed using a yeast two-hybrid system and co-immunoprecipitations (Kaiser, 2006). Taken together, the findings suggest that the non-structural proteins NS2, NS4, and the helicase assist in the formation of replication complexes in FCV-infected cells.

In MNV infections, NS1/2 localizes to the ER while NS4 localizes to the Golgi (Table 1). Nevertheless, both NS1/2 and NS4 are associated with replication complexes. To study the involvement of NS4 in replication, an infectious MNV variant with a FLAG-tagged NS4 protein was generated using transposon-mediated insertional mutagenesis (Thorne et al., 2012). Immunofluorescence staining revealed co-localization of NS4 with the RdRp, and co-immunoprecipitation showed an interaction of NS4 with NS1/2. This demonstrates that both NS1/2 and NS4 were present at the site of MNV replication (Thorne et al., 2012). Furthermore, intracellular localization studies with specific marker proteins suggest that active MNV replication complexes contain membranous vesicles derived from the ER, medial- and trans-Golgi apparatus, and endosomes (Hyde et al., 2009). Thus, the role of the NS1/2 and NS4 proteins in this process is believed to be recruiting ER and Golgi membranes, respectively (Hyde and Mackenzie, 2010; Thorne et al., 2012). To further characterize the viral replication machinery, Hosmillo et al. (2019) used infectious MNV variants with a FLAG tag on either NS1/2 or NS4 for immunoprecipitations of active replication complexes. The researchers found that these complexes contained all viral proteins, and interestingly, a number of cellular proteins associated with fatty acid metabolism and vesicular transport, such as the protein VAP-A (Hosmillo et al., 2019).

### Viroporin Activity

Viroporins are viral proteins that feature one, two, or three transmembrane α-helices (Hyser et al., 2010). The helices possess amphipathic properties that allow for efficient membrane incorporation: hydrophobic amino acid residues face the membrane, and polar residues line the pore. These proteins oligomerize to form a functional transmembrane ion channel, which can be selective or non-selective and voltage dependent or independent (reviewed in Nieva et al., 2012). For example, the influenza virus protein M2 has only one transmembrane helix; however, through the formation of tetramers, enough transmembrane helices are brought together to form a small pore that is selectively permeable to protons (Pinto et al., 1997). A remarkable function mediated by many viroporins is the disruption of cellular Ca^2+^ homeostasis through leakage from intracellular depots (mitochondria, ER, Golgi) to the cytoplasm (Aldabe et al., 1997; Pham et al., 2017). Changes to the intracellular Ca^2+^ concentration can favor viral replication and induce apoptosis (Hajnóczky et al., 2003; Zhou et al., 2009).

The picornavirus protein 2B has multiple functions including that of a viroporin. In poliovirus-infected cells, 2B oligomerizes and forms ion channels in ER and Golgi membranes (Martinez-Gil et al., 2011). When expressed as recombinant proteins, 2B and interestingly, also 3A disrupted Ca^2+^ signaling, suggesting that polioviruses encode two viroporins that act independently (Doedens and Kirkegaard, 1995; Aldabe et al., 1997; Martinez-Gil et al., 2011).

Similar activities were described for calicivirus non-structural proteins. For example, the Tulane virus NS1/2 was shown to form an ion channel in ER membranes and to elevate cytoplasmic Ca^2+^ levels (Strtak et al., 2019). Its ability to form an ion channel was initially predicted *in silico* by identifying amphipathic transmembrane helices in the C-terminal (NS2) part of NS1/2. The functionality of the domain has since been confirmed *in vitro* using a “classic” bacterial viroporin assay. This assay takes advantage of a genetically modified *Escherichia coli* strain that constitutively expresses low levels of T7 lysozyme to control T7 RNA polymerase-dependent gene expression. The low-level expression of the lysozyme is normally well tolerated, but co-expression of proteins that form membrane channels/pores can lead to leakage of the lysozyme into the periplasmic space, cleavage of peptidoglycan, and the subsequent lysis of the cell (Studier and Moffatt, 1986). The expression of full-length NS1/2, but not that of several deletion mutants, caused cell lysis in T7 lysozyme-producing *E. coli* (Strtak et al., 2019). Furthermore, eukaryotic cells stably expressing a fluorescent Ca^2+^ sensor were infected with *Tulane virus* and the intensity of fluorescence was measured over time. After 8 h, the cytoplasmic Ca^2+^ concentration in virus-infected cells was significantly higher than that in mock-infected cells. Moreover, when Ca^2+^ levels were depleted in the cell culture medium and in the cytoplasm using the Ca^2+^ chelator BAPTA-AM [1,2-bis(o-aminophenoxy)ethane-N,N,N',N'-tetraacetic acid], virus replication decreased dramatically, suggesting that Ca^2+^-mediated signaling is crucial for the Tulane virus life cycle (Strtak et al., 2019).

**Figure 4:**
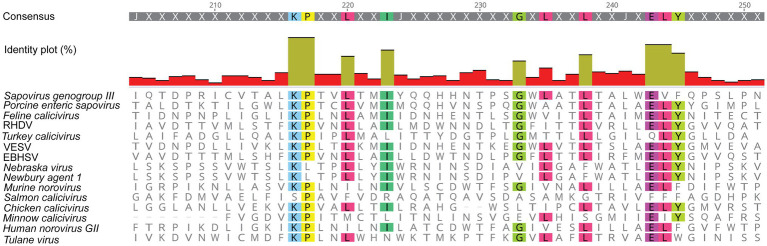
Amino acid sequence alignment of the putative viroporin domain in the NS2 protein of various caliciviruses. The C-terminal region of all NS2 proteins contains a high degree of conserved amino acid residues (highlighted in color), as compared to the rest of the protein sequence. This figure was generated using the same sequences as in Figure 2B. RHDV, *Rabbit haemorrhagic disease virus*; EBHSV, *European brown hare syndrome virus*; and VESV, *Vesicular exanthema of swine virus*.

It becomes increasingly clear that similar viroporins exist among all caliciviruses. In 2003, Ettayebi and Hardy (2003) identified a hydrophobic transmembrane domain in the NS1/2 of human and murine noroviruses (Figure 3), even though it was not clear at the time that this domain is part of a viroporin. Furthermore, the C-terminal region of all NS2 proteins shows a remarkable degree of conservation among caliciviruses – relative to other parts of the protein (Figure 4). In noroviruses, the C-terminal part of the NS1/2 protein includes a number of relatively hydrophobic regions (HRs) that form a distinct secondary structure, whereas most of the N-terminal is largely hydrophilic and disordered (i.e., a secondary structure is lacking; Baker et al., 2012). The C-terminal hydrophobic transmembrane domain (along with upstream sequences) is responsible for the observed co-localization of the human norovirus NS1/2 with Golgi membranes in transfected cells and is essential for the disassembly of the Golgi apparatus (Fernandez-Vega et al., 2004). When a fusion construct of the hydrophobic domain of NS1/2 (without upstream sequences) and the green fluorescent protein was expressed, it co-localized with the Golgi but did not disrupt its membranes. Thus, sequences upstream of the HR are required for Golgi disruption (Fernandez-Vega et al., 2004). In another experiment, transposon-based insertional mutagenesis was used to probe MNV genome tolerance for a 15-nt exogenous sequence (Thorne et al., 2012). In the C-terminal region of NS2, most of these 15-nt insertions were lost after three passages, suggesting that this part of the protein is required for virus replication (Thorne et al., 2012). Moreover, functional assays measuring intracellular Ca^2+^ levels revealed that the NS1/2 of human norovirus also disrupts calcium homeostasis, similar to the Tulane virus experiments (Strtak et al., 2019).

**Figure 5:**
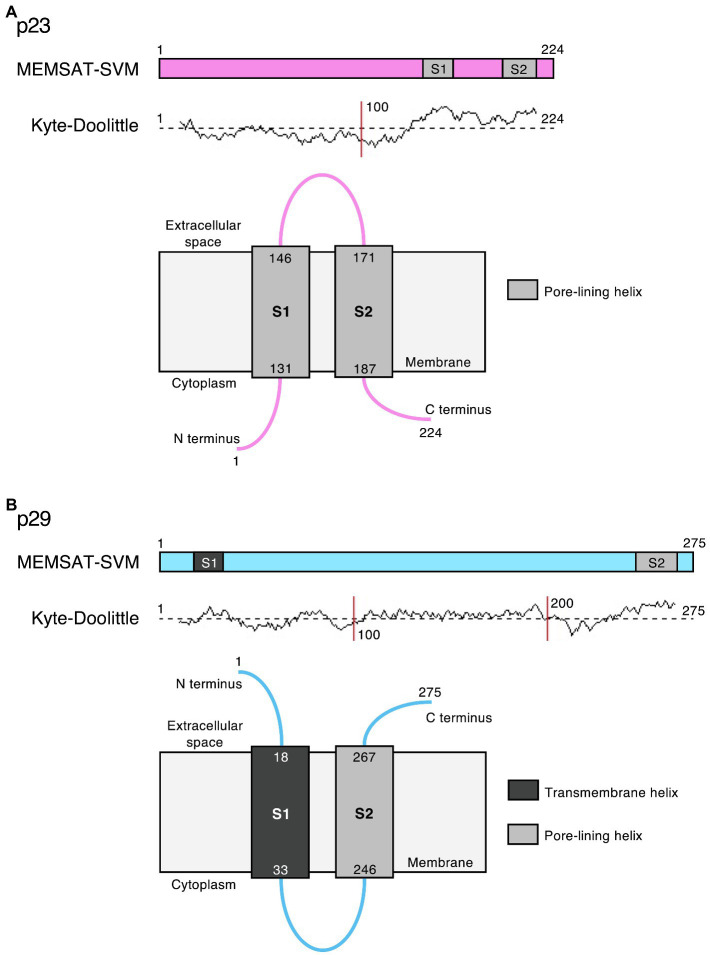
Potential viroporin domains in the lagovirus (RHDV) non-structural proteins. Sequence analysis using PSIPRED secondary structure prediction tools (Buchan and Jones, 2019) revealed transmembrane (dark gray) and/or amphipathic pore-lining helices (light gray) in **(A)** p23 (pink) and **(B)** p29 (light blue); the MEMSAT-SVM algorithm (Nugent and Jones, 2012) was used for protein topology prediction; and Kyte-Doolittle plots indicate the hydrophobicity of amino acids. Note that the exact intracellular localization of p23 and p29 and the orientation of these proteins in cellular membrane(s) is currently unknown; if they localize to the ER, the term “cytoplasm” would indicate the lumen of the organelle.

Bioinformatic predictions suggest that the lagovirus proteins NS2 and NS4 contain amphipathic helices that may interact with membranes to form ion channels and possibly act as viroporins, similar to the transmembrane domains in picornavirus homologs 2B and 3A, respectively (Figure 5). This hypothesis is further supported by the observation that RHDV NS2 oligomerizes in transfected cells (Urakova et al., 2015), which would bring together a sufficient number of transmembrane helices to form a functional viroporin. However, functional studies are needed to confirm this hypothesis.

## Counteracting Innate Immune Responses

Poliovirus protein 2A (a chymotrypsin-like protease) counteracts the IFN-induced antiviral defense: wild-type poliovirus can efficiently replicate in cells pre-treated with IFN-α, while a poliovirus variant with a mutation in 2A that affects the cleavage of cellular but not viral substrates, no longer replicated in IFN-treated cells (Morrison and Racaniello, 2009). The exact mechanism for this phenomenon is not known. Not all picornaviruses have 2A proteins that counteract the IFN system, e.g., the 2A of *Encephalomyocarditis virus* (EMCV) seems to lack the ability to interfere with innate immune responses. The replication of EMCV is IFN-sensitive; the virus does not replicate in cells pre-treated with IFN-α. However, IFN resistance can be engineered by substituting the EMCV 2A gene with the gene of its poliovirus homolog, which further demonstrates the importance of 2A in counteracting innate immune responses (Morrison and Racaniello, 2009). Other poliovirus non-structural proteins have additional immune evasion functions, e.g., the proteins 2B and 3A inhibit the secretion of IFN-β, proinflammatory interleukins (IL)-6 and 8, and the intracellular trafficking of the receptor for the tumor necrosis factor (TNF; Dodd et al., 2001; Neznanov et al., 2001).

The calicivirus proteins NS1/2 and NS4 are likely to be homologs of the picornavirus proteins 2A/2B and 3A, respectively (see Figure 1). Evidence is accumulating that all of these proteins are involved in the downregulation of host defense responses. The norovirus protein NS1 is cleaved from its precursor protein NS1/2 by caspase-3 (a cellular protease) and is “secreted” through an unconventional pathway (Lee et al., 2019). Extracellular NS1 seems to be essential for overcoming epithelial defenses induced by IFN-λ (Lee et al., 2019). A recombinant MNV variant with a mutation in the caspase-3 cleavage site of NS1/2 failed to replicate in wild-type mice after oral infection, although replication upon intraperitoneal injection was unaffected (Lee et al., 2019). Virus replication after oral infection was rescued in type III IFN receptor deficient but not in type I IFN or type II IFN receptor deficient mice (Lee et al., 2019), suggesting that type III IFNs such as IFN-λ play a critical role in epithelial host defenses against noroviruses. The importance of a type III IFN-mediated antiviral response was further demonstrated in human intestinal organoids (enteroids). Human norovirus-infected enteroids responded to the infection by expressing type III but not type I IFNs (Lin et al., 2020). In additional experiments, genetically targeted enteroid lines were used to determine whether knocking out key IFN signaling components would enhance virus replication. Interestingly, Lin et al. (2020) found that the replication of a bile acid-dependent GII.3 strain but not that of a pandemic GII.4 strain was increased in enteroid lines without a functional IFN type I/IFN-α/β receptor (IFNAR) or the latent transcription factor STAT1. However, a similar increase in virus replication was not observed in genetically targeted enteroids that no longer expressed STAT2 *and* STAT1, or in enteroids without a functional IFN type III receptor (Lin et al., 2020). Thus, additional research is needed to fully understand the role of the different IFNs and IFN-induced effector proteins in norovirus infections. Nevertheless, the observed strain-specific sensitivities to innate immune responses may help to identify norovirus proteins with an ability to counteract IFN signaling and/or IFN-induced effector proteins.

Manipulating the host cell’s transcriptional activity is a way through which many viruses counteract host immune responses. A transcriptome analysis of transiently transfected monocytes revealed that norovirus NS1/2 reduces the expression of toll-like receptor (TLR)-4, -7, -8, and -9, increases the expression of several pro-inflammatory cytokines/chemokines, and induces a pro-apoptotic phenotype, suggesting that the norovirus NS1/2 protein regulates innate and adaptive immune responses (Lateef et al., 2017). In addition, norovirus NS1/2 may manipulate innate immune responses at the protein level. The aforementioned interaction between the norovirus NS1/2 and the vesicle-associated membrane protein VAP-A suggests that caliciviruses manipulate intracellular trafficking, which would inhibit or block the transport of critical innate immune proteins to the cellular surface (TLRs, IFNs, MHCs, etc.).

Interestingly, in FCV, it is the NS4 (p30) protein (i.e., the picornavirus 3A homolog) that interferes with host cell innate immune responses. Tian et al. (2020) analyzed the IFN signaling in FCV-infected cells that were pre-treated with the transcription inhibitor actinomycin D (to stop virus-induced transcription). When they analyzed the mRNA levels of the IFN-α/β receptor subunits 1 and 2 (IFNAR1 and IFNAR2, respectively), they found that the half-life of IFNAR1 but not IFNAR2 mRNAs was drastically reduced in virus-infected cells compared to control cells (6.3 vs. 100 h, respectively). This showed that FCV downregulates expression of a functional IFN type I receptor through mRNA degradation of IFNAR1 (Tian et al., 2020). To identify the protein responsible for the IFNAR1 mRNA degradation, cells were transiently transfected with each of the FCV non-structural proteins and the IFNAR1 mRNA concentrations were measured. NS4 was the only protein that significantly affected IFNAR1 mRNA stability (Tian et al., 2020).

The picture emerges that caliciviruses have evolved various mechanisms to counteract innate immune defenses. It is likely that even more counter defense mechanisms exist, but their discovery is currently hampered by a lack of robust cell culture models and replicon systems.

## Miscellaneous Features

Enteroviruses are well-known for their ability to shutoff host cell protein expression (Barco et al., 2000). This function is largely attributed to the viral proteases 2A and 3C; both proteases process viral polyproteins, but can also cleave several host proteins (Ventoso et al., 1998; Agol and Gmyl, 2010). For example, 2A manipulates the nuclear pore by cleaving one of the nuclear pore components, thereby inhibiting mRNA export (Belov et al., 2004; Castelló et al., 2009). Furthermore, 2A and 3C attack the poly(A) binding protein (PABP; Joachims et al., 1999) and 2A cleaves a component of the translation initiation factor eIF4F, which effectively stops translation of capped mRNAs (Kräusslich et al., 1987). Caliciviruses also interfere with the cellular protein expression, but the mechanistic details are less clear. The viral 3C-like proteases of human noroviruses, MNV and FCV were shown by several researchers to be associated with translational shutoff and cleavage of PABP, similar to poliovirus proteases (Kuyumcu-Martinez et al., 2004; Emmott et al., 2017). Other researchers did not find an involvement of the MNV 3C-like protease but observed that cellular protein synthesis was inhibited by NS3 (the helicase) through an unknown mechanism (Fritzlar et al., 2019). Clearly, future research is warranted to resolve this discrepancy.

Interestingly, the 2A proteins of enteroviruses and the NS1/2 protein of caliciviruses possess an H-NC motif (Figure 3). This motif contains an H-box with a characteristic histidine amino acid residue and an NC motif with an asparagine and cysteine dipeptide (Hughes and Stanway, 2000; Johansson et al., 2002; Fernandez-Vega et al., 2004). Why these non-structural proteins have a conserved H-NC motif is unknown, but the occurrence of the motif in a cellular protein may give some clues to its function. The class II tumor suppressor protein H-rev107 is a phospholipase that also possesses the H-NC motif (Hughes and Stanway, 2000; Uyama et al., 2009). Through its ability to bind K-Ras, H-rev107 inhibits cell growth and differentiation, and regulates apoptosis (Han et al., 2017, 2020). It is possible that viral proteins such as 2A and NS1/2 manipulate key signaling proteins like K-Ras, but there is presently no evidence to support this hypothesis.

## Outlook

Until now, research into the non-structural proteins of caliciviruses has mostly been focused on MNV and FCV (mainly because these viruses can be cultivated easily in a laboratory). New organoid culture systems, e.g., human (Ettayebi et al., 2016) and rabbit intestinal organoids (Mussard et al., 2020; Kardia et al., 2021) may soon facilitate detailed functional studies on the replication of human noroviruses, lagoviruses, and other caliciviruses that do not grow in conventional cell culture. These studies will also shed light on the role of non-structural proteins in building replication complexes, counteracting innate and adaptive immune responses, redirecting cellular resources, and other activities.

## Author Contributions

ES developed the conceptual outline and drafted the manuscript. ES, RH, NU, TS, and MF wrote the manuscript and contributed to editing and revising the manuscript. All authors contributed to the article and approved the submitted version.

### Conflict of Interest

The authors declare that the research was conducted in the absence of any commercial or financial relationships that could be construed as a potential conflict of interest.
